# What kind of science for psychiatry?

**DOI:** 10.3389/fnhum.2014.00435

**Published:** 2014-06-20

**Authors:** Laurence J. Kirmayer, Daina Crafa

**Affiliations:** Division of Social and Transcultural Psychiatry, McGill University & Institute of Community and Family Psychiatry, Jewish General HospitalMontreal, QC, Canada

**Keywords:** critical neuroscience, psychiatric diagnosis, nosology, research domain criteria, culture, social context, systems science

## Abstract

Psychiatry has invested its hopes in neuroscience as a path to understanding mental disorders and developing more effective treatments and ultimately cures. Recently, the U.S. NIMH has elaborated this vision through a new framework for mental health research, the Research Domain Criteria (RDoC). This framework aims to orient mental health research toward the discovery of underlying neurobiological and biobehavioral mechanisms of mental disorders that will eventually lead to definitive treatments. In this article we consider the rationale of the RDoC and what it reveals about implicit models of mental disorders. As an overall framework for understanding mental disorders, RDoC is impoverished and conceptually flawed. These limitations are not accidental but stem from disciplinary commitments and interests that are at odds with the larger concerns of psychiatry. A multilevel, ecosocial approach to biobehavioral systems is needed both to guide relevant neuroscience research and insure the inclusion of social processes that may be fundamental contributors to psychopathology and recovery.

## INTRODUCTION

Psychiatry has invested its hopes in neuroscience as a path to understanding mental disorders and developing more effective treatments and ultimately cures. Recently, the U.S. NIMH has elaborated this vision through a new framework for mental health research, the Research Domain Criteria (RDoC; Insel, 2013). This framework aims to re-orient mental health research toward the discovery of underlying neurobiological and biobehavioral mechanisms of mental disorders that will eventually lead to definitive treatments. Does this strategy make sense? Will it bear fruit? Are there any reasons to be concerned about the scope and limits of this type of research program? In this article we consider the rationale of the RDoC and what it reveals about implicit models of mental disorders. We argue that the RDoC presents a useful scheme for advancing current neurobiological research along established lines. As an overall framework for understanding mental disorders, however, it is impoverished and conceptually flawed. These limitations are not accidental but stem from disciplinary commitments and interests that are at odds with the larger concerns of psychiatry.

## WHAT IS A MENTAL DISORDER?

Assessing the strengths and limitations of a comprehensive research program in psychiatry depends on clarifying the types of problems or conditions that psychiatry aims (or is mandated) to understand and treat. If psychiatry is the medical discipline or helping profession concerned with mental disorders, circumscribing the boundaries of psychiatry depends on having some notion of what counts as a mental disorder. Unfortunately, efforts to do this have foundered on the great diversity of problems seen by psychiatrists as well as the fuzzy borders or boundaries between health, normal variations, and pathology.

The problems included in current psychiatric nosologies [Diagnostic and Statistical Manual (DSM-5) and International Classification of Disease (ICD-10)] include a great variety of afflictions that represent very different sorts of problems. This way of partitioning problems is the result of a 150-year history in which psychiatry emerged from neurology and the custodial care of the chronic mentally ill to encompass a much wider range of conditions that overlap with some of the most common problems of everyday life. No single definition can capture this broad and heterogeneous group of problems that includes many different families of disorders that likely will turn out to involve very different underlying mechanisms. The common element across this diversity is some disturbance in higher or complex functions of thinking, feeling, behavior, and experience. Of course, this begs the question of how to distinguish disturbed or abnormal functioning from normal variations in these systems.

Normality as a biomedical construct or category may be construed in at least three different ways that rest on different kinds of knowledge: (i) what is statistically common or average in a population; (ii) what is part of the adaptive functioning of an organism in a given ecological environment; and (iii) what is normatively prescribed or expected in a particular social and cultural context. Applying each of these notions of normality to brain functioning raises its own epistemological and methodological difficulties. However, all three definitions are clearly context-dependent. Moreover, although these different ways of defining normality may be inter-related (for example, there is some expectation that in a healthy population what is most frequently found will correspond to what is most adaptive), in general, these forms of normality need not be in register with each other.

A basic issue raised by efforts to define mental disorders concerns establishing the boundaries between normality and pathology (Cooper and Sartorius, 2013). Categorical schemes tend to treat this as a binary or dichotomous determination: a person either has or does not have (any or a specific) mental disorder. Normality can then be defined simply as the absence of any pathology. Yet many symptoms exist on a spectrum or continuum from mild expressions that might be viewed as variants of normality through to severe symptoms associated with impairment. While it is possible to measure impairment in broad terms across domains of everyday activity or social role functioning, different types of mental health problems may differentially affect specific aspects of functioning and particular domains of life. Hence, global ratings of functioning cannot adequately capture what counts as a mental health problem. Moreover, it is precisely the difficulty in coping with symptoms or problems (e.g., chronic pain, anxiety, or dysphoric mood) that may make them into disabling conditions for which people seek clinical help.

In an effort to clarify the boundaries of what counts as a mental disorder, Wakefield (1992, 2007) has argued that we can distinguish the normative part of the general notion of disorder, which depends on social norms and judgments, from the functional part which reflects some deviation or disturbance of the usual (i.e., intended, designed, healthy) functioning of a system. However, many critics have pointed out the difficulty in defining function completely free of social context and norms so that in most cases Wakefield’s disarticulation fails (Lilienfeld and Marino, 1995; Kirmayer and Young, 1999; Murphy and Woolfolk, 2000). The ways we use our brains and the kinds of functions we need to adapt to a particular environment depend on specific social and cultural contexts, histories, and developmental trajectories. Hence, values are woven into our efforts to define adaptive functions.

We can learn something useful from the difficulty defining mental health and illness in abstract, global terms: mental disorders, both individually and in the aggregate, depend in fundamental ways on meaning and social context. This reflects not only the dynamics of problem recognition, labeling, and social response but something intrinsic to the sorts of problems we view as psychological or psychiatric. Mental functioning in health and in illness involves inhabiting, adapting, and responding to the demands of local social worlds. This has implications for any approach to neuroscience in psychiatry that aims to be clinically relevant.

## THE DSM AS A CONCEPTUAL FRAMEWORK IN PSYCHIATRIC RESEARCH

Although the ICD-10 is the official nosology globally authorized by treaties, the DSM of the American Psychiatric Association is the dominant diagnostic system in North America and has become a de facto standard internationally in part because it includes useful accompanying text but especially because it has played a dominant role in training and research. For over 30 years, the DSM system has shaped not only the practice of psychiatry but also the research enterprise. Most studies funded by NIMH and other agencies have been framed in terms of discrete diagnostic groups defined by the DSM.

While earlier versions of U.S. psychiatric nosology were underwritten by psychoanalysis, DSM-III ushered in an era of operationalizing diagnostic categories through specific symptom criteria (Wilson, 1993). DSM-III was driven by a medical model that saw psychiatric disorders as closely analogous to physical diseases. The approach promoted by Eli Robins and colleagues at Washington University in St. Louis emphasized characteristics they believed would identify discrete psychiatric disorders or diseases, including: (1) consistent clinical descriptions of syndromes based on symptoms, signs, and behaviors; (2) laboratory tests consistently associated with the syndrome; (3) family aggregation; (4) relationship to course and outcome; and (5) specificity, that is ability to distinguish different types of problems (Robins and Guze, 1970; Goodwin and Guze, 1974). This methodological strategy was underwritten by a kind of biological essentialism that assumed that psychiatric disorders would turn out to be discrete biological entities, each with its own distinctive causes and pathophysiological mechanisms. The criterion of family aggregation, for example, was based on the assumption that genetic factors play an important role in psychiatric disorders―though family aggregation could occur for other reasons related to shared environment (Kendler, 2006), family interaction, and learning history, or even a shared narrative that influences family members’ expectations and styles of coping (Fivush et al., 2011).

In addition to aligning psychiatry with the rest of medicine, there was the hope that distinctions could be made between disorders that had implications for differential therapeutics; specifically, the recognition of differences in symptoms and course between schizophrenia and manic depressive (bipolar) disorder which were important for prognosis, became still more important because of differential treatment (e.g., neuroleptics versus lithium). Thus, findings in psychopharmacology gave credibility and urgency to efforts to distinguish discrete groups of mental disorders. The results of the US/UK study showing that bipolar disorder was under-diagnosed in the US in favor of schizophrenia became an important impetus to reform diagnostic practices (Cooper et al., 1972). The success of antidepressant medications in treating major depressive disorder warranted its consistent recognition and diagnosis, which was sometimes obscured by psychodynamic approaches that focused on cross-cutting personality traits, character structure, and defense mechanisms (Klerman, 1990; Strand, 2011). The conviction that DSM-III was on the right track rested on these few important examples of differential diagnosis associated with differential prognosis and therapeutics.

Over time, however, the expected refinements of DSM-III did not materialize. Syndromes proved to be much less discrete than intended, with very high rates of co-occurrence or “comorbidity.” This was interpreted as evidence of a failure to define discrete disorders, although this comorbidity may reflect not simply overlap in the symptoms used to define disorders, or the misfortune of a person struck by two disorders (certainly a possibility for disorders with high prevalence rates), but the existence of causal mechanisms that link one disorder to another through specific symptom-related processes (e.g., prolonged anxiety causing depression; Borsboom et al., 2011).

Potential laboratory tests emerged but lacked the necessary specificity and sensitivity. There was a shift from surface, “phenotypic” characteristics to a search for “endophenotypes,” underlying physiological expressions of pathology that could be measured through biomarkers (Kendler and Neale, 2010; Kapur et al., 2012). The search for biomarkers that would uniquely characterize specific disorders yielded interesting findings but none had sufficient specificity for clinical use. In particular, the search for discrete genetic characteristics has turned up a great many genes with some association to psychiatric disorders but, in most cases, these are neither necessary nor sufficient causes of any particular symptom or disorder. Instead, it appears that many genetic variations make multiple, small, cumulative contributions to risk for many different types of disorder. Moreover, recognition of the complexity of the dynamic regulation of the genome over the lifespan has given rise to new fields of epigenomics and developmental systems theory (Champagne, 2010; Zhang and Meaney, 2010).

Treatments also have proved to have far less specificity than originally thought (Healy, 1997, 2004; Paris and Phillips, 2013). Antidepressants work for panic disorder, obsessive-compulsive disorder, and many other conditions. Neuroleptics work for psychotic symptoms of many origins and increasingly have been employed as mood stabilizers and augmenting agents for various non-psychotic conditions (e.g., OCD). To some extent, this wide use follows marketing efforts by pharmaceutical companies to extend (and over-extend) medications to new conditions, but it also suggests that the therapeutic efficacy of medications reflects their effects on common pathways or systems involved in symptom production not necessarily involved in mechanisms specific to particular forms of psychopathology.

DSM-III provided a much-needed increase in the clarity with which clinicians communicated and precision in diagnostic definitions. Much of this clarity and precision, however, was achieved by adopting symptom criteria, thresholds, and exclusion rules with “pseudo-precision” (e.g., including specific numbers or duration of symptoms in criteria based on limited studies). The DSM has been a serviceable tool for psychiatric assessment in those areas where diagnosis is reliably linked to prognosis and treatment. At the same time, there has been growing concern that reliability (that is consistent application of criteria) was bought at the price of validity (correspondence with distinct forms of clinically relevant psychopathology).

The DSM has also exerted a profound effect on psychiatric research because studies funded by the U.S. NIMH and other major funding bodies have often been framed in terms of its diagnostic entities. To the extent that the use of DSM diagnostic categories has been a requirement, whether explicitly built into funding programs or as part of tacit ways of thinking, research has contributed to a process of reification in which the accrual of evidence about the constructs makes them seem more solid and natural, if not inevitable, and impedes the development of new constructs (Hyman, 2010).

As time went on, it became apparent that the categories of DSM-III were not related in any simple way to underlying neurophysiological mechanisms. As a result, research progress in refining the diagnostic symptom was slow and DSM-IV was a relatively conservative revision and DSM-IV-TR mainly a matter of minor revisions to the text.

## THE PROMISE AND FAILURE OF DSM-5

Preliminary work on DSM-5 began in the late 1990s and there was initial hope that the DSM-5 would usher in a nosology driven by neuroscience (Hyman, 2007; Paris and Phillips, 2013). A series of research planning workshops was held to set out a broad agenda (Kupfer et al., 2002). A subsequent set of meetings summarized available evidence across diagnostic domains. Much consideration was given to the introduction of dimensional rather than categorical schemes as reflecting the reality that many symptoms occurred along a continuum, which together formed a multidimensional space in which various clusters of problems could be located (Helzer et al., 2008). Dimensions were supported by substantial psychometric evidence in several cases (e.g., personality traits that might underlie personality disorders). The dimensional approach was touted as better reflecting biological reality. In some cases, there was sufficient research evidence from animal models and human functional brain imaging to speak in terms of specific brain circuitry. For example, a meeting was held on stress-related syndromes and fear circuitry pointing toward a reorganization of anxiety and trauma-related disorders (Andrews, 2009; Simpson, 2012).

However, when the time came to formulate the revised diagnostic system, the scientific evidence proved insufficient to guide and justify major changes. In some cases, vested interests (in terms of theoretical schools or the exigencies of clinical practice) trumped scientific evidence. More generally, practical clinical considerations were raised against a dimensional approach to diagnosis, which was viewed as unwieldy and as simply deferring the necessary decisions (label or not, treat or not). As a result, DSM-5 was a fairly conservative revision (Regier et al., 2013). Even in the area of personality disorders, where there was strong evidence from psychometric research for the value of a dimensional approach, the decision was ultimately to retain a categorical scheme similar to that of DSM-IV (Livesley, 2013). Much disappointment has been expressed with the outcome and there is a sense of deflation and skepticism about the whole enterprise (Paris and Phillips, 2013; Whooley, 2014). Ironically, one of the more innovative aspects of DSM-5 concerns the introduction of a cultural formulation interview designed to contextualize diagnosis (Lewis-Fernández et al., 2014), but this does not directly address the issue of putting the structure of the nosology and its categories on a firmer footing by grounding it in evidence about etiology and mechanism.

## ENTER THE RDoC: RE-ORDERING PSYCHIATRIC RESEARCH IN THE U.S.

In terms of basic science research, one critique of the DSM centers on the failure to find distinct neurobiological correlates for most disorders. This has been interpreted as a failure to adequately define the phenotype; that is, the DSM is accused of creating overly heterogeneous or spurious categories that do not have single, simple neurobiological mechanisms; hence, the search for underlying mechanisms is futile. Of course, it is possible that the existing categories have some validity (e.g., at the level of patient’s experience, predictions of prognosis, differential therapeutics) but that the pathways from etiology, through mechanism to symptoms are complex, multifactorial, and do not admit a neurobiological explanation in terms of a small number of causal factors and mechanisms. Decomposing the diagnostic categories into underlying mechanisms and re-constituting a nosology based on identifiable interactions among component systems might result in diagnostic constructs with greater coherence and utility. However, if complex interactions among multiple systems give rise to psychopathology, it will be difficult or impossible to assign unique causality to any single mechanism (Mitchell, 2009). Nosology would then have to reflect not the component systems but emergent patterns of interaction among them. Moreover, the fact that most current diagnostic categories are heterogeneous may not reflect limitations in the nosology but the intrinsic complexity of mechanisms of psychopathology, in which there may be final common pathways of symptom production and experience relevant to multiple forms of pathology. Moving the search for mechanism back several steps in the causal chain to putative endophenotypes may increase the likelihood of finding certain lower-level mechanisms but it will not provide a complete explanation of how most symptoms are produced nor will it adequately address the role in psychopathology of processes of self-understanding, coping, and interpersonal communication or interaction with others.

To advance the search for underlying mechanisms, the U.S. NIMH has developed its own scheme, the RDoC, and has parted ways with the APA very publically and polemically (Insel, 2013). In contrast to the clinical orientation of the DSM-5, the RDoC offers a research framework that relies on functional domains, defined by preliminary research on brain circuitry (Insel et al., 2010; Cuthbert and Insel, 2013). In its initial version, RDoC includes five major domains covering negative valence systems (i.e., those that respond to aversive situations), positive valence systems, cognitive systems, systems for social processes, and arousal/regulatory systems. Much like the DSM, these domains of research were established by roundtable discussions among leading experts. As such, they represent a snapshot of current work in neurobiology. The list of domains and constructs is expected to change and expand with new research.

**FIGURE 1:**
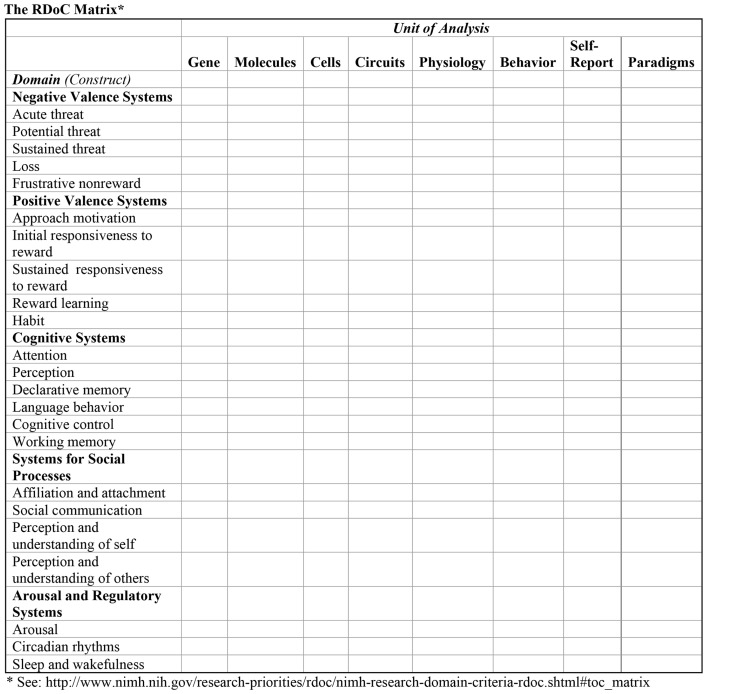
**The NIMH RDoC framework organized as a 2 × 2 matrix with rows for broad biobehavioral domains that group together specific constructs and columns representing levels of analysis, units, or types of data**. Neural circuitry is the central level of analysis on the assumption that behavioral and experiential manifestations of psychopathology can be traced to underlying circuitry which, in turn, can be analyzed in terms of cellular, molecular, and genetic mechanisms. Rows may be further subdivided in terms of specific constructs. A column is reserved for experimental paradigms, which may be specific to domains.

The RDoC is a dimensional framework intended to advance research and produce a new diagnostic system based on biological measures, particularly neurobiology. The RDoC is presented as 2 × 2 matrix with the rows representing *domains* and *constructs* and the columns *units of analysis* (**Figure 1**). The domains group together constructs that represent behavioral functions for which there is some knowledge of underlying neurobiological mechanisms. The units of analysis represent the methodological tools, measures, and levels of analysis currently available for research on “normal” or adaptive functioning as well as psychopathology. It is not clear to what extent the two dimensions are independent in the sense that the units or levels of analysis are largely driven by the domains and constructs which, in turn, emphasize areas where there are currently available models of brain circuitry.

The primary focus of RDoC is on neural circuitry, with levels of analysis moving in two directions: upward from physiological measures of the circuitry to clinically relevant behaviors and self-reports, and downward to the genetic and molecular/cellular processes that underlie the structure and function of brain circuits. A core assumption of RDoC is that mental disorders are brain disorders, specifically disorders of brain circuits and that dysfunction of these circuits can be identified with the tools of neuroscience (electrophysiology, functional imaging, connectomics), which will lead to etiological models of mental disorders and to curative treatments that directly target these mechanisms (Insel, 2013). Although this may be a long-term goal, the framers of RDoC believe that heavy investment in this program is warranted since research strategies with more immediate return will not lead to curative treatments but merely stop-gap methods of managing symptoms.

## DILEMMAS OF THE RDoC FRAMEWORK

The virtues of the RDoC approach are related to its breaking away from the rigid diagnostic categories of existing psychiatric nosology and include: (1) an emphasis on the potential continuity or links between “normal” adaptive functioning and psychopathology; (2) a focus on potential mechanisms for psychopathology that builds on existing experimental paradigms (especially animal models that allow a wide range of invasive experimental techniques to examine neural circuitry, cellular, and molecular machinery); and (3) a commitment to remain open to new dimensions, constructs, and levels of analysis. In particular, the RDoC architects recognize the importance of the temporal dimension, particularly in terms of developmental trajectories and processes of gene–environment interaction, including epigenetics (Cuthbert, 2014, p. 30). They also acknowledge the importance of the environment, although this is discussed mainly in terms of the influence of physical circumstances or discrete events like childhood trauma on biological processes.

Despite the potential value of its approach, there are many criticisms of and concerns about RDoC as a comprehensive framework for guiding research on mental health problems. Here we will focus on three broad sets of problems with RDoC: (1) the limitations of approaching pathology through “normal” mechanisms; (2) the privileging of the level of neural circuitry and comparative lack of attention to levels of explanation that would include social, interpersonal, and other processes; and (3) the relative lack of attention to phenomenology, narrativity, and lived experience.

## THE NORMAL AND THE PATHOLOGICAL

The Research Domain Criteria assumes that dysfunction can be best understood against the backdrop of normal functioning and frames this in terms of a set of questions: “What is the normal distribution for a certain trait or characteristic; what is the brain system that primarily implements this function; and, how can we understand, at various levels of mechanism, what accounts for the development of dysregulation or dysfunction in these systems along normal-to-abnormal dimensions?” (Cuthbert, 2014, p. 31). Understanding psychopathology in terms of variations of normality has many merits. An understanding of normal functioning may prevent pathologizing ordinary variations and point to how usually adaptive processes may become part of vicious circles that result in pathology. Interpreting the results of brain imaging or other neurobiological measures depends on having a clear sense of how these structures and processes usually function. Of course, this begs the question of how one establishes norms. Identifying dysfunction or dysregulation of a functional system requires assumptions about normal functioning that are not independent of context. Although the examples of psychopathology targeted by proponents of RDoC tend to be major neuropsychiatric disorders that have similar symptomatology across cultures and contexts, most psychiatric disorders show substantial individual, cultural, and contextual variation (Gone and Kirmayer, 2010). In choosing to focus on disorders that show less variation or that have animal models, RDoC-driven research would inevitably set aside many of the most common mental health problems. While norms might be defined against adaptive niches and physiological parameters in animals, they will generally have to be recalibrated and reconsidered to reflect human functioning in specific environmental, social, and cultural contexts that vary widely.

Ironically, the road to pathology via normality creates its own problem of heterogeneity. To illustrate how RDoC would encourage cross-cutting measurements of circuitry, Cuthbert suggests that “samples for a study of reward circuit activity (as relevant to anhedonia and/or mania) might be drawn from virtually the entire population of treatment- seeking adults – mood/anxiety spectrum, psychotic spectrum, eating disorders, personality disorders; for appropriate exploration of dimensionality, the sample would also include relatively minor psychopathology such as an adjustment reaction diagnosis as well as those individuals who do not meet criteria for any diagnosis” (Cuthbert, 2014, p. 32). After critiquing the DSM for creating heterogeneous categories that do not map onto discrete neurocircuitry, it seems inconsistent if not incoherent to emphasize looking for dysfunction across such disparate phenotypes. The assumption seems to be that the underlying mood-regulation circuits do much the same thing no matter what the neural and behavioral contexts within which they operate. In terms of the context of the brain, insofar as circuits are influenced (perhaps even reorganized) by their input from other neural processes, or the larger circuits in which they are embedded, this hope may be unfounded. In terms of the larger context of adaptation, individuals with particular types of psychopathology and social predicaments, may have to use their brains differently to adapt and this could lead to very different patterns of activity in circuits.

The focus on normality also may ignore the unique qualities of pathology (David, 2010). There is a long tradition in the philosophy of biology and medicine, arguing that pathology has its own characteristics and requires its own methods of study (Canguilhem, 1989; Tiles, 1993). The clinico-pathological method collects cases that exemplify extreme forms of disorders in order to understand their characteristics. The assumption is that pathology may have its own unique features and, moreover, that the mechanisms of “breakdown” may sometimes be easier to discern than those of normal functioning. If this method has failed so far to yield specific neurobiological correlates for most psychiatric disorders, it may be because the alterations in functional systems are subtler or reflect the interaction of multiple systems that subserve complex behavior (Mitchell, 2009).

If pathology has its own unique characteristics that are not simply the extreme end of statistically defined normality, the search for mechanisms of psychopathology cannot be limited to the study of “normal” systems because the ways that neural systems break down may reflect the dynamics of pathology, including distinctive changes in structure and function as well as the ways that various pathogenic agents challenge, perturb, and disrupt neural circuitry and processes. The dynamics of pathology may include various states of neural systems that are not active ordinarily but reached only through pathological developmental trajectories or histories of learning. Even the effects of pathogenic physical factors agent may involve distinctive physiological derangements; e.g., toxins, mutations, injuries, infectious agents all have their own characteristics and dynamics distinct from those of the adaptive system. As a result, as has long been recognized in medicine, pathology is a study in its own right. However limited our current models of adaptive processes, side-stepping or postponing the study of pathology by focusing primarily on normal functioning is going to limit or delay understanding the mechanisms of illness and disease.

## THE SEARCH FOR ENDOPHENOTYPES AND BIOSIGNATURES

In place of a focus on examining psychopathology, the RDoC program adopts a “translational research” approach in which well-studied neurobiological mechanisms of normal functioning are translated into clinical models and interventions (Casey et al., 2013; Insel and Landis, 2013). The conviction is that data from genetic and clinical neuroscience will yield “biosignatures” (the language implies something more specific than a biomarker, the biological equivalent of a pathognomonic sign) that can complement, augment and, in some cases, ultimately replace clinical diagnoses based on symptoms and signs. Of course, this approach cannot completely set aside the phenomenological categories and social predicaments that are the focus of clinical work. Clinical work involves translation back and forth from one framework (neurobiology) to another (clinical presentations) that is articulated in terms of behavior and experience. If endophenotypes replace behavior and experience, we may end up with a situation in which the biologically defined parameters are assessed and treated while the patient is asked to stand to one side. The RDoC model is that of physical medicine, where a clinician may diagnose and attempt to treat diabetes regardless of the patient’s recognition and understanding of the disease. Of course, effective treatment requires the patient’s engagement and active collaboration but the focus of technical expertise in biomedicine tends to be on the disease rather than the person. Indeed, this tendency to displace the person by the disease is precisely what many have critiqued in current biomedicine. The remedy on offer of more research on neurobiological mechanisms does not contribute directly to efforts to develop a person-centered medicine (Mezzich et al., 2010) – as distinct from a personalized medicine that focuses on tailoring treatment to individuals’ biological differences – and, if pursued to the exclusion of other levels of explanation that include individuals’ illness experience and lifeworlds, will work against efforts to humanize care.

By focusing on constructs with established neurobiology, the RDoC matrix assumes that existing research areas are a good place to start the discovery process. Mining at the exposed coal face makes sense in terms of immediate productivity (and support for established researchers with productive paradigms) but it is an investment in “normal science” and, depending on how it is deployed in research programs, may discourage more exploratory studies aimed at developing entirely new constructs.

Although current brain research methods have important limitations, they are undergoing constant refinement. For example, much of the neurobiological research supporting the RDoC includes fMRI studies, which often yield inconsistent and tenuous findings (Vul et al., 2009; Ramsey et al., 2010; Todd et al., 2013). Current techniques lack sufficient temporal and anatomical resolution to answer fine-grained questions about process. Further, the differences in blood flow and metabolism measured in functional MRI need not have a simple correspondence to specific neural functioning (Shulman, 2013). As a body of work is built up mapping function onto specific pathways, new studies will be easier to interpret and will yield new insights. Ultimately, however, the problems with fMRI research may not be just the technical limitations of the method but the assumptions that guide the formulation of research questions and hypotheses: it is possible that psychopathology does not lie in functional specific neural processes alone; rather, psychopathology may arise from larger system dynamics (Kirmayer and Gold, 2012). The brain adapts to a social world and its modes of function and dysfunction can best be understood in relation to the opportunities and demands afforded by specific social contexts and positions.

The RDoC scheme assumes that underlying biological mechanisms will be similar across species and individuals. This is framed as a rejection of “human exceptionalism”: human beings are animals like any other as reflected in “the surprising conservation of genes, neurotransmitters, and behavioral functions across evolution – even in model animals such as fruit flies and zebrafish” (Cuthbert, 2014, p. 31). Hence, a rodent model of depression, autism or schizophrenia can stand in for human psychopathology on the assumption that the underlying mechanisms are not only relatively homogeneous across individuals, populations, and cultures but also across species. Indeed, there is such confidence in the universality of mechanisms that one type of study envisages bringing together symptomatically diverse groups of people to be studied in terms of the same construct. This puts a lot of faith in the stability or similarity of neural systems functioning in the context of very different forms of psychopathology. Yet it is possible that people vary individually and culturally in ways that not only change some parameters within a given circuit but that actually alter the functions of that circuit in relation to the larger organization of behavior. Moreover, it is possible that pathology itself results in different modes of functioning so that certain processes are simply not seen at the mild or “normal” end of the spectrum. Far from revealing the links between normal functioning and pathology, therefore, including a wide spectrum of patients in the same study may obscure the changing meaning of neural function in the context of specific types of disorder (Kagan, 2012). As Weinberger and Goldberg (2014) point out, the way that a person with autism or schizophrenia responds to the laboratory setting of an fMRI study may differ markedly from someone without this type of psychopathology, violating a basic assumption of experimental design and complicating the interpretation of any observed differences.

Moreover, neural responses may vary across individuals according to their sociocultural background and the immediate social context, as well as other factors, like affective state, tacit values, and intentions which may be mobilized by specific contextual cues or task demands (e.g., Kim and Sasaki, 2014; Ma et al., 2014). Findings from patients with one social background or in one social context therefore may not be generalizable to populations with similar symptoms. While symptoms of underlying disorders may vary across individuals, apparently similar symptoms may have different causes, which change their dynamics and implications for clinical prognosis and treatment. All of these arguments apply not only to overt symptoms but to endophenotypes as well.

## LEVELS OF EXPLANATION: CIRCUITS, SYSTEMS, AND CONTEXTS

RDoC uses the term “neural circuitry” to describe a level of analysis but this must be seen as a metaphor for certain kinds of models of brain functioning, since most current methodology only allows indirect measurement of putative underlying neural circuits. In practice, the circuits currently studied in neuroscience vary from detailed description of small networks of neurons in animals derived from single unit recording, through anatomical and functional mapping of large white matter tracts (the “connectome;” Sporns, 2013), to box-and-arrow diagrams of information processes based on anatomical localization of activation during specific tasks. Convergence among these and other methodologies can clarify the nature of information processing in the brain; however, the definition and meanings of “circuitry” differ in each of these cases, in terms of the components of the circuit, their dynamics, and the associated levels of processing.

The early versions of neural network theory represented a limit case of extreme simplicity. The fact that it was possible to construct a universal Turing machine from such simple constituents (McCulloch and Pitts, 1943) served to show the computational possibilities and gave impetus to the development of neural network models with greater biological reality. In reality, with over 1000 different types of neurons in the brain and with potential emergent phenomena of ephaptic transmission and synchronous firing through local electromagnetic fields, the nervous system is immensely more powerful than such simple networks. More complex networks have their own emergent properties that have not yet be sufficiently considered in the simple box-and-arrow diagrams of information processing models of cognitive neuroscience (Alivisatos et al., 2012). Indeed, the boxes or nodes in neural systems models represent functional modules that integrate multiple inputs to produce output that participates in larger systems. These modules have their own dynamics that interact with the larger system dynamics.

Although circuits are portrayed as discrete, isolable systems that can be analyzed without reference to any larger system, the biological reality is that they are nested within larger systems. The brain as a whole provides a context for individual circuits or networks that perform multiple functions that can only be fully understood in terms of the overall system. Hence, the functions of individual circuits may only be understood when they are viewed in the context of the larger networks in which they participate. Beyond the individual brain, the social world provides contexts that have shaped neural organization and functioning on multiple timescales. Culture can be seen as providing essential contexts for the development and functioning of the brain on multiple timescales: through its evolutionary history, which has involved brain–culture coevolution; across individual lifespans as biographical events are inscribed in circuitry by mechanisms of epigenetics and learning; and through ongoing influences on neural functioning by specific contexts of adaptation and performance (Krubitzer and Stolzenberg, 2014). The emerging field of cultural neuroscience is showing how the observed differences between ethnocultural groups in behavior and experience are associated with corresponding changes in neural functioning in response to specific tasks or contexts (Han et al., 2013; Wang et al., 2013; Kim and Sasaki, 2014). Interpreting these results requires understanding the meanings of culture for the individual in terms of developmental trajectories, social roles, skills, and the expectations, goals, and strategies evoked by a given social context (Choudhury and Kirmayer, 2009; Seligman et al., in press). While these social, cultural, and contextual dimensions of behavior and experience are reflected in the architecture and functioning of the brain, they remain located in the social world in institutions, discourse, and practices.

Explications of RDoC mention the importance of a third dimension of time, representing developmental processes and trajectory, and a fourth dimension of environment. Unfortunately, the developmental and contextual dimensions of RDoC have not been elaborated to the same extent as the neurobiological constructs or levels of analysis. Indeed, close attention to development and to environmental and social contexts would lead to a different set of levels of analysis that encompass interpersonal, social, and cultural contexts (Kirmayer and Gold, 2012). These levels are relevant not only to research on social constructs but may provide novel insights into the nature of biological mechanisms. To take an example close to Insel’s own area of research, the neurohormone oxytocin, which has been found to be associated with pair bonding and feelings of trust, may not simply activate such prosocial systems, but make individuals more sensitive to social cues (Bartz et al., 2011). Characterizing the behavioral effects of oxytocin may then require a deeper understanding of kinds of social situations and relationships and the tradeoffs that may occur for individuals living in communities that are very different in size and structure than the evolutionary contexts in which the neurohormonal system originally emerged (Kim and Sasaki, 2014).

Attention to the environment not as physical or material space but as fundamentally social contexts, situations or predicaments would encourage consideration of other key constructs and measures (Kirmayer and Gold, 2012). In turn, this would lead to novel ways to approach the nature of psychopathology in specific domains. Methodologies that are widely used to study social context, including ethnography and narrative-based research have no obvious place in the RDoC scheme – presumably because they are not easily associated with specific brain circuitry but, perhaps, also because of a deeper skepticism about the scientific utility of studying subjectivity and experience.

## NARRATIVE, ACTION, AND EXPERIENCE

A final set of issues raised by RDoC arises from the fact that many complex, experiential aspects of psychopathology cannot be studied with animal models. By emphasizing animal models in research, distinctively human processes are sidelined. Central among these are language and narrativity. The human ability to construct stories as vehicles for cognition and communication is important for many aspects of psychological conflict, coping, and healing.

Since the time of Jaspers there has been recognition that illness experience and clinical presentations reflect both neuropathology and the patient’s attitude toward the illness (Stanghellini et al., 2013). Even the most severe forms of psychopathology owe their experiential and behavioral manifestations not just to their underlying biology but also to cognitive-interpretive and interpersonal processes that are at play prior to the onset of illness, during the genesis of symptoms, and throughout the course of illness. These processes are not incidental to psychopathology but intrinsic to its “natural history,” which is necessarily also a personal and social history, mediated by meaning-centered processes. Chief among these are the embodied and enacted processes of metaphor and the linguistic practices of narrative.

Although patients’ self-descriptions are viewed in current psychiatry primarily as ways to identify symptoms to assign a diagnosis, the metaphors, and narratives that people use to understand and communicate their experience contribute to the underlying dynamics of illness. Narratives can be causal mechanisms in human experience (and psychopathology) in several different ways: (i) by influencing cognitive processes involved in the regulation of attention and the interpretation of sensory experience and perception. For example, attributional processes are central to symptom experience and guide coping, help-seeking and treatment response (Kirmayer and Bhugra, 2009); (ii) by configuring the structure of goals, plans or intentions in terms of feedback loops, with set-points in behavior that constitute plans or goals of action (Miller et al., 1960; Juarrero, 1999). This builds on an essential insight of cybernetics that adaptive systems are structured in terms of regulatory feedback systems (Wiener, 1948/1961; Ashby, 1960; Arbib, 1972; Pickering, 2010), but adds the notion that the comparator functions or set-points in these systems can be set up by cognitive processes that are governed by narratives; (iii) by giving rise to or constraining other narratives. Narratives are generative and have their own dynamics of coherence and creativity, reflected in processes such as dissonance reduction and imaginative elaboration (Oatley, 1992). These narrative processes may be sources of suffering and of adaptation (Lewis, 2011); (iv) by positioning the person in a social world, communicating and eliciting specific responses from others. This is the social action perspective (Kleinman, 1988); and (v) by leading to or evoking larger narrative structures and discursive formations in which others participate – and which reframe and given new meaning to their actions. These larger social narratives may shape individual experience in self-vindicating loops (Hacking, 1999, 2002). This is an important process in psychiatry because diagnostic constructs tend to become part of popular discourse and modes of self-understanding.

Although many social processes occur through non-verbal interactions across the lifespan, narrative represents a particular important bridge between individual psychological processes and the social world. As such, the study of narrative processes – both in terms of individual cognition, interpersonal interaction and wider social/discursive processes – is vital to understanding key mental health processes including symptom interpretation, coping, regulation of self-efficacy and self-esteem, and stigma.

## EMBODIED BRAINS, ENACTED MINDS: TOWARD SYSTEMS NEUROSCIENCE FOR PSYCHIATRY

Despite hope for neurobiological explanations for mental disorders, models to date are speculative, with insufficient empirical support, and do not lead to clear predictions in terms of prognosis or treatment, nor, indeed, to a clear nosology dividing one type of problem from another. Despite intensive search, there are no clinically useful biomarkers for psychopathology. There are various ways to interpret this failure.

It may be that we simply have too little neurobiological data. Mental disorders are complex and varied and we need to accumulate much more data until the key variables can be identified and the causal patterns emerge. Recent projects that aim to accelerate current research by building large datasets partly reflect this assumption but also take advantage of new methods in bioinformatics to search for patterns. Genomics has used whole genome array studies to search for correlates of psychiatric disorders. This same method can be extended to endophenotypes with more success. Moreover, a similar program focused on the human connectome (white matter tracts and pathways of functional connectivity) may arrive at novel endophenotypes. However, these approaches still require theoretical models of functional systems to know which of the many correlations are meaningful in terms of potential mechanisms and which are random variation or “noise.”

It might also be that we are collecting the wrong kind of neurobiological data. We need to look at other levels or areas than those currently studied. In particular, it might be that the complexity of mappings from causal processes to phenotypes involves so many alternate pathways at each level that the correlations become small and hard to detect. If so, then working out ways to partition problems based on more refined clinical and cross-cultural phenomenology will remain an important strategy.

Finally, it is possible that there are inherent limitations of the conceptual project of neurobiological explanation. The search for mechanisms in the brain makes strong assumptions about how human behavior and psychopathology emerges that may not be warranted for every type of problem included within the purview of psychiatry.

Neuroscience increasingly recognizes that what is going on for the brain is not only in the brain but also in the body and in the social world (Walter, 2013). Much recent work in cognitive science has argued that the mind is “extended,” meaning that it is embodied, enacted, situated, and distributed in the environment and, especially, the social world (Varela et al., 1991; Clark and Chalmers, 1998; Menary, 2010; Rowlands, 2010).

Just as biological processes influence the brain’s responses, potentials, and limitations, sociocultural influences play a large role in human brain development and processing across the lifespan. Under extreme circumstances, this environmental influence can severely constrain subsequent biological development. For example, infants kept in prolonged social isolation exhibit abnormal brain development and fail to acquire basic motors skills or language (Hildyard and Wolfe, 2002; Perry, 2002; Lin et al., 2005; Watts-English et al., 2006; Rees, 2008). The social environment profoundly influences epigenetic and endocrine processes (Cole, 2009; Champagne, 2010; Curley et al., 2011) as well as the neural circuitry involved in psychiatric disorders, including such higher functions as language (Paulesu et al., 2000) and person perception (Turner, 2002; Freeman and Ambady, 2014). In each case, a dynamic interplay between social and biological processes results in clinical symptoms, impairments in functioning, and persistent illness or recovery. The interactions of brain and environment occur on many levels simultaneously. Together, brain, body, and the social environment form a mutually regulatory adaptive system (Crafa and Nagel, in press).

The brain itself is a complex, hierarchical system in which layers or levels of sensory, affective, and cognitive processing acquired over evolutionary history are integrated into flexible functioning to subserve the adaptive organization of behavior. Although multi-level systemic concepts of the brain have been proposed in the past, the focus of most neuroscience in psychiatry has been on the biological mechanics of neural functioning at synaptic, cellular, and molecular levels. In most cases, this involves examining linear causal processes by isolating or abstracting simpler models from the larger systems in which they are embedded. In so doing, some of the systems dynamics crucial to understanding psychopathology may be lost (Henningsen and Kirmayer, 2000).

The theory of cybernetics introduced by Wiener (1948/1961) emphasized the role of “feedback loops” in the regulation of behavior. While focused on organism–environment systems, this approach provides a way to think about the influence of the social world on internal (neural or cognitive) processes. Higher cognitive processes, including consciousness, can then be understood as control mechanisms in human behavior that emerged from and subsequently influence evolutionary processes (Bateson, 1972). In psychiatry, this cybernetic perspective spawned family systems theory and therapy, which showed how certain mental health problems emerge vicious circles in interpersonal interaction. This systems approach has been viewed by many as distant from (or irrelevant to) the biological nuts and bolts of psychopathology and the effort to explain drug treatments in terms of specific molecular, genetic or neurophysiological mechanisms. However, advances in neural network theory and systems neuroscience point toward renewed applications of dynamical systems theory and computational modeling in psychiatry (Montague et al., 2012). These conceptual frameworks and tools will be essential to make sense of the large bodies of data generated by neuroscience by enabling data reduction, pattern recognition, and model testing. Such systems thinking can also provide new approaches to a psychiatric nosology grounded in a typology of vicious circles that includes psychological, interpersonal, and wider social processes (Kirmayer and Sartorius, 2009).

## CONCLUSION: METHODOLOGICAL PLURALISM AND THE SYSTEMIC VIEW

Clearly, RDoC is a vision of the future. Given the rudimentary nature of data relating measures of brain function to psychopathology, it may take a long time before clinically useful tools are produced. Although there are already efforts to apply its decomposition of domains to a step-wise approach to psychotherapy (e.g., Alexopoulos and Arean, 2014), evidence for the efficacy of this type of modularized intervention is limited. Nevertheless, NIMH views RDoC as the beginning of a transformative effort over the next few decades toward implementing a neuroscience-based psychiatric classification and approach to treatment. To the extent that RDoC stimulates new and creative thinking it may be an important step in the evolution of psychiatric science. Seen as one component of the NIMH portfolio of mental health research, RDoC represents an innovative approach to understanding the nature of psychiatric disorders. If it narrows the research program of NIMH to exclude major avenues of research, however, there is much cause for concern about its impact.

RDoC may define its notion of circuits and other levels and systems constructs broadly enough to include the kinds of studies that can capture context sensitivity and non-linear dynamics. Examining how the RDoC criteria evolve as the program is implemented is an interesting problem for sociology of science. Analyzing the intrinsic limitations of the conceptual framework and determining what sorts of paradigms must be included to capture the dynamics of mental disorders poses an interesting set of questions for the philosophy of psychiatry (Kirmayer, 2012). It is possible that, even with very broad definitions, the RDoC framework imposes constraints on the kind of studies likely to be funded that will distort, limit, and delay or prevent understanding of essential mechanisms. In particular, the RDoC framework would seem to prefer studies that are: (1) experience-distant, setting aside behavioral and experiential phenotypes for endophenotypes; (2) focus on mechanisms that cut across disorders or clinical problems and presentations, rather than those that are intrinsic to specific types of problems; and (3) favor explanations in terms of linear causality in circuits depicted described in terms of anatomical loci linked in box and arrow flow charts, rather than the distributed networks and regulatory systems that cannot be anatomically localized and that have crucial causal links and components outside the individual person.

In parallel with RDoC, other major initiatives that seek to map and model the whole brain or large-scale connectivity will offer a refined anatomical picture that may lend itself to a richer model of circuitry. But this will require comparable advances in our modeling of brain function to make sense of its connectivity. Just as the human genome project explained much less than was hoped but provided a toolkit with which to begin to unravel specific gene–protein-function correspondences and pointed toward whole new notions of mechanism, so too the human brain project will likely provide us not with simple explanations of psychopathology in terms of single causes but with new tools to explore the interactions of brain and environment. Systems biology and neuroscience are developing tools to model the emergent dynamics of complex networks that can help us go beyond the linear causal models that currently dominate both research and clinical thinking (Tretter and Gebicke-Haerter, 2009; Alivisatos et al., 2012; Montague et al., 2012). Crucially, however, these systems approaches must be applied not only to the isolated circuit, module or brain, but to the person in ecosocial context.

In sum, a multilevel, systemic approach with expanded consideration of social context would have many benefits both for guiding the development of a comprehensive research program and for developing a nosology with greater clinical utility. We currently have some of the tools needed to implement such systems-oriented research. Mixed-methods approaches that combine research paradigms can reveal links between social and biological events. This can be as simple as assessing changes in a biological parameter in specific types of salient social situations (defined in terms of their meaning for the person and their entourage) or as complex as mapping the systems dynamics of dyads or families organized by historical narratives, cultural norms, and longstanding relationships as well as ongoing interaction. In this kind of multilevel research, the coherence of the paradigm can come from its personal and social meaning rather than its reducibility to a discrete neural circuit.

Identifying system dynamics can provide clinicians with new ways to assess problems in terms of feedback loops with circular causality (Kirmayer and Sartorius, 2009). These loops may depend crucially on social contexts, so that we need a nosology based not just on neural circuits but on personal and social predicaments. The choice of level of explanation and intervention then will be based not on the unwarranted assumption that molecular or circuitry levels are more fundamental but on pragmatic decisions about where clinical leverage can be found. Interventions at any level may have impact throughout the hierarchical system, with causal influence working both bottom-up and top-down. From this perspective, it is as meaningful to ask what changes are wrought in the brain in response to trusting relationships as it is to ask how a specific change in amygdala function influences social interaction (Phan et al., 2006; Lupien et al., 2011). The fact that these multilevel influences work in both directions means that insight into the nature of healthy functioning as well as psychopathology is as likely to come from understanding social and interpersonal dynamics as it is from looking at brain circuitry. This recognition would move psychiatric theory, research, and practice beyond the search for discrete biological mechanisms toward understanding and treating the whole person in the context of family, community, and lifeworld.

## Conflict of Interest Statement

The authors declare that the research was conducted in the absence of any commercial or financial relationships that could be construed as a potential conflict of interest.

## References

[B1] Alexopoulos G. S. Arean P. (2014). A model for streamlining psychotherapy in the RDoC era: the example of ‘Engage’. *Mol. Psychiatry* 19 14–19 10.1038/mp.2013.150 24280983 PMC4337206

[B2] Alivisatos A. P. Chun M. Church G. M. Greenspan R. J. Roukes M. L. Yuste R. (2012). The brain activity map project and the challenge of functional connectomics. *Neuron* 74 970–974 10.1016/j.neuron.2012.06.006 22726828 PMC3597383

[B3] Andrews G. (ed.). (2009). *Stress-Induced, and Fear Circuitry Disorders: Advancing the Research Agenda for DSM-V*. Arlington, VA: American Psychiatric Association

[B4] Arbib M. A. (1972). *The Metaphorical Brain: An Introduction to Cybernetics as Artificial Intelligence and Brain Theory*. New York: Wiley-Interscience 10.1002/gps.930060318

[B5] Ashby W. R. (1960). *Design for a Brain*. London: Chapman & Hall, Ltd

[B6] Bartz J. A. Zaki J. Bolger N. Ochsner K. N. (2011). Social effects of oxytocin in humans: context and person matter. *Trends Cogn. Sci.* 15 301–309 10.1016/j.tics.2011.05.002 21696997

[B7] Bateson M. C. (1972). *Our Own Metaphor: A Personal Account of a Conference on the Effects of Conscious Purpose on Human Adaptation*. New York: A.A. Knopf

[B8] Borsboom D. Cramer A. O. J. Schmittmann V. D. Epskamp S. Waldorp L. J. (2011). The small world of psychopathology. *PLoS ONE* 6:e27407 10.1371/journal.pone.0027407 PMC3219664 22114671

[B9] Canguilhem G. (1989). *The Normal and the Pathological*. New York: Zone Books

[B10] Casey B. J. Craddock N. Cuthbert B. N. Hyman S. E. Lee F. S. Ressler K. J. (2013). DSM-5 and RDoC: progress in psychiatry research? *Nat. Rev. Neurosci*. 14 810–814 10.1038/nrn3621 24135697 PMC4372467

[B11] Champagne F. A. (2010). Epigenetic influence of social experiences across the lifespan. *Dev. Psychobiol*. 52 1–13 10.1002/dev.20436 20175106

[B12] Choudhury S. Kirmayer L. J. (2009). Cultural neuroscience and psychopathology: prospects for cultural psychiatry. *Prog. Brain Res.* 178 261–281 10.1016/S0079-6123(09)17820-2 PMC5161496 19874976

[B13] Clark A. Chalmers D. (1998). The extended mind. *Analysis* 58 7–19 10.1093/analys/58.1.7

[B14] Cole S. W. (2009). Social regulation of human gene expression. *Curr. Dir. Psychol. Sci*. 18 132–137 10.1111/j.1467-8721.2009.01623.x 21243077 PMC3020789

[B15] Cooper J. E. Kendell R. E. Gurland B. J. Sharpe L. Copeland J. R. M. Simon R. (1972). *Psychiatric Diagnosis in New York and London*. London: Oxford University Press

[B16] Cooper J. E. Sartorius N. (2013). A Companion to the Classification of Mental Disorders. New York, NY: Oxford University Press

[B17] Crafa D. Nagel S. K. (in press). Traces of culture: the feedback loop between brain, behavior, and disorder. *Transcult. Psychiatry*. 10.1177/1363461519879515 31996101

[B18] Curley J. P. Jensen C. L. Mashoodh R. Champagne F. A. (2011). Social influences on neurobiology and behavior: epigenetic effects during development. *Psychoneuroendocrinology* 36 352–371 10.1016/j.psyneuen.2010.06.005 20650569 PMC2980807

[B19] Cuthbert B. N. (2014). The RDoC framework: facilitating transition from ICD/DSM to dimensional approaches that integrate neuroscience and psychopathology. *World Psychiatry* 13 28–35 10.1002/wps.20087 24497240 PMC3918011

[B20] Cuthbert B. N. Insel T. R. (2013). Toward the future of psychiatric diagnosis: the seven pillars of RDoC. *BMC Med.* 11:126 10.1186/1741-7015-11-126 PMC3653747 23672542

[B21] David A. S. (2010). Why we need more debate on whether psychotic symptoms lie on a continuum with normality. *Psychol. Med.* 40 1935 10.1017/S0033291710000188 20624330

[B22] Fivush R. Bohanek J. G. Zaman W. (2011). Personal and intergenerational narratives in relation to adolescents’ well-being. *New Dir. Child Adolesc. Dev.* 2011 45–57 10.1002/cd.288 21387531

[B23] Freeman J. B. Ambady N. (2014). “The dynamic interactive model of person construal: coordinating sensory and social processes,” in *Dual Process Theories of the Social Mind* edsSherman J. Gawronski B Trope Y. New York: Guilford Press

[B24] Gone J. P. Kirmayer L. J. (2010). “On the wisdom of considering culture and context in psychopathology,” in *Contemporary Directions in Psychopathology: Scientific Foundations of the DSM-V and ICD-11* edsMillon T. Krueger R. F. Simonsen E. (New York: Guilford) 72–96

[B25] Goodwin D. Guze S. (1974). *Psychiatric Diagnosis*. New York: Oxford University Press

[B26] Hacking I. (1999). *The Social Construction of What?* Cambridge, MA: Harvard University Press

[B27] Hacking I. (2002). *Historical Ontology*. Cambridge, MA: Harvard University Press

[B28] Han S. Northoff G. Vogeley K. Wexler B. E. Kitayama S. Varnum M. E. (2013). A cultural neuroscience approach to the biosocial nature of the human brain. *Annu. Rev. Psychol.* 64 335–359 10.1146/annurev-psych-071112-054629 22994921

[B29] Healy D. (1997). *The Antidepressant Era*. Cambridge: Harvard University Press

[B30] Healy D. (2004). *The Creation of Psychopharmacology*. Cambridge: Harvard University Press 10.1177/0957154X04045686 15272483

[B31] Helzer J. E. Kraemer H. C. Krueger R. F. Wittchen R. F. Sirovatka P. J. Regier D. A. (eds). (2008). *Dimensional Approaches in Diagnostic Classification: Refining the Research Agenda for DSM-V*. Arlington, VA: American Psychiatric Association

[B32] Henningsen P. Kirmayer L. J. (2000). Mind beyond the net: implications of cognitive neuroscience for cultural psychiatry. *Transcult. Psychiatry* 37 467–494 10.1177/136346150003700401

[B33] Hildyard K. L. Wolfe D. A. (2002). Child neglect: developmental issues and outcomes. *Child Abuse Negl.* 26 679–695 10.1016/S0145-2134(02)00341-1 12201162

[B34] Hyman S. E. (2007). Can neuroscience be integrated into the DSM-V? *Nat. Rev. Neurosci*. 8 725–732 10.1038/nrn2218 17704814

[B35] Hyman S. E. (2010). The diagnosis of mental disorders: the problem of reification. *Annu. Rev. Clin. Psychol.* 6 155–179 10.1146/annurev.clinpsy.3.022806.091532 17716032

[B36] Insel T. (2013). *Transforming Diagnosis. NIMH Director’s Blog.* Available at: http://www.nimh.nih.gov/about/director/index.shtml

[B37] Insel T. Cuthbert B. Garvey M. Heinssen R. Pine D. S. Quinn K. (2010). Research domain criteria (RDoC): toward a new classification framework for research on mental disorders. *Am. J. Psychiatry* 167 748–751 10.1176/appi.ajp.2010.09091379 20595427

[B38] Insel T. R. Landis S. C. (2013). Twenty-five years of progress: the view from NIMH and NINDS. *Neuron* 80 561–567 10.1016/j.neuron.2013.09.041 24183009 PMC3859529

[B39] Juarrero A. (1999). *Dynamics in Action: Intentional Behavior as a Complex System*. Cambridge, MA: MIT Press.

[B40] Kapur S. Phillips A. G. Insel T. R. (2012). Why has it taken so long for biological psychiatry to develop clinical tests and what to do about it? *Mol. Psychiatry* 17 1174–1179 10.1038/mp.2012.105 22869033

[B41] Kagan J. (2012). *Psychology’s Ghosts: The Crisis in the Profession and the Way Back*. New Haven: Yale University Press

[B42] Kendler K. (2006). Reflections on the relationship between psychiatric genetics and psychiatric nosology. *Am. J. Psychiatry* 163 1138–1146 10.1176/appi.ajp.163.7.1138 16816216

[B43] Kendler K. S. Neale M. C. (2010). Endophenotype: a conceptual analysis. *Mol. Psychiatry* 15 789–797 10.1038/mp.2010.8 20142819 PMC2909487

[B44] Kim H. S. Sasaki J. Y. (2014). Cultural neuroscience: biology of the mind in cultural contexts. *Annu. Rev. Psychol.* 65 487–514 10.1146/annurev-psych-010213-115040 24050186

[B45] Kirmayer L. J. (2012). “The future of critical neuroscience,” in *Critical Neuroscience: A Handbook of the Social and Cultural Contexts of Neuroscience* edsChoudhury S. Slaby J. (Oxford: Blackwell) 367–383

[B46] Kirmayer L. J. Bhugra D. (2009). “Culture and mental illness: social context and explanatory models,” in *Psychiatric Diagnosis: Patterns and Prospects* edsSalloum I. M. Mezzich J. E. (New York: John Wiley & Sons) 29–37

[B47] Kirmayer L. J. Gold I. (2012). “Re-socializing psychiatry: critical neuroscience and the limits of reductionism,” in *Critical Neuroscience: A Handbook of the Social and Cultural Contexts of Neuroscience* edsChoudhury S. Slaby J. (Oxford: Blackwell).

[B48] Kirmayer L. J. Sartorius N. (2009). “Cultural models and somatic syndromes,” in *Somatic Presentations of Mental Disorders: Refining the Research Agenda for DSM-V* edsDimsdale J. E. Patel V. Xin Y. Kleinman A. Sirovatka P. J. Regier D. A. (Washington: American Psychiatric Press) 23–46

[B49] Kirmayer L. J. Young A. (1999). Culture and context in the evolutionary concept of mental disorder. *J. Abnorm. Psychol.* 108 446–452 10.1037/0021-843X.108.3.446 10466268

[B50] Kleinman A. (1988). *The Illness Narratives*. New York: Basic Books

[B51] Klerman G. L. (1990). The psychiatric patient’s right to effective treatment: implications of Osheroff v. Chestnut Lodge. *Am. J. Psychiatry* 147 409–418 1969242 10.1176/ajp.147.4.409

[B52] Krubitzer L. Stolzenberg D. S. (2014). The evolutionary masquerade: genetic and epigenetic contributions to the neocortex. *Curr. Opin. Neurobiol.* 24 157–165 10.1016/j.conb.2013.11.010 24492091

[B53] Kupfer D. J. First M. B. Regier D. A. (2002). *A Research Agenda for DSM-V*. Washington, DC: American Psychiatric Association

[B54] Lewis B. (2011). *Narrative Psychiatry: How Stories Can Shape Clinical Practice*. Baltimore: Johns Hopkins University Press

[B55] Lewis-Fernández R. Aggarwal N. K. Bäärnhielm S. Rohlof H. Kirmayer L. J. Weiss M. G. (2014). Culture and psychiatric evaluation: operationalizing cultural formulation for DSM-5. *Psychiatry* 77 130–154 10.1521/psyc.2014.77.2.130 24865197 PMC4331051

[B56] Lilienfeld S. O. Marino L. (1995). Mental disorder as a Roschian concept: a critique of Wakefield’s “harmful dysfunction” analysis. *J. Abnorm. Psychol.* 104 411–420 10.1037/0021-843X.104.3.411 7673564

[B57] Lin S. H. Cermak S. Coster W. J. Miller L. (2005). The relation between length of institutionalization and sensory integration in children adopted from Eastern Europe. *Am. J. Occup. Ther*. 59 139–147 10.5014/ajot.59.2.139 15830613

[B58] Livesley J. (2013). The DSM-5 personality disorder proposal and future directions in the diagnostic classification of personality disorder. *Psychopathology* 46 207–216 10.1159/000348866 23652353

[B59] Lupien S. J. Parent S. Evans A. C. Tremblay R. E. Zelazoi P. D. Corbo V. (2011). Larger amygdala but no change in hippocampal volume in 10-year-old children exposed to maternal depressive symptomatology since birth. *Proc. Natl. Acad. Sci. U.S.A*. 108 14324–14329 10.1073/pnas.1105371108 21844357 PMC3161565

[B60] Ma Y. Bang D. Wang C. Allen M. Frith C. Roepstorff A. (2014). Sociocultural patterning of neural activity during self-reflection. *Soc. Cogn. Affect. Neurosci.* 9 73–80 10.1093/scan/nss103 22956678 PMC3871729

[B61] McCulloch W. S. Pitts W. A. (1943). A logical calculus of the ideas immanent in nervous activity. *Bull. Math. Biophys.* 5 115–133 10.1007/BF02478259 2185863

[B62] Menary R. (2010). *The Extended Mind*. Cambridge, MA: MIT Press 10.7551/mitpress/9780262014038.001.0001

[B63] Mezzich J. E. Salloum I. M. Cloninger C. R. Salvador-Carulla L. Kirmayer L. J. Banzato C. E. M. (2010). Person-centered integrative diagnosis: conceptual basis and structural model. *Can. J. Psychiatry* 55 701–708 21070697 10.1177/070674371005501103

[B64] Miller G. A. Galanter E. Pribram K. H. (1960). *Plans and the Structure of Behavior*. New York: Holt, Rinehart and Winston 10.1037/10039-000

[B65] Mitchell S. D. (2009). *Unsimple Truths: Science, Complexity, and Policy*. Chicago: University of Chicago Press 10.7208/chicago/9780226532653.001.0001

[B66] Montague P. R. Dolan R. J. Friston K. J. Dayan P. (2012). Computational psychiatry. *Trends Cogn. Sci.* 16 72–80 10.1016/j.tics.2011.11.018 22177032 PMC3556822

[B67] Murphy D. Woolfolk R. L. (2000). The harmful dysfunction analysis of mental disorder. *Philos. Psychiatry Psychol.* 7 241–252

[B68] Oatley K. (1992). “Integrative action of narratives,” in *Cognitive Science and Clinical Disorder* edsStein D. J. Young J. E. (San Diego: Academic Press) 151–170

[B69] Paris J. Phillips J. (eds). (2013). *Making the DSM-5: Concepts and Controversies*. New York: Springer 10.1007/978-1-4614-6504-1

[B70] Paulesu E. McCrory E. Fazio F. Menoncello L. Brunswick N. Cappa S. F. (2000). A cultural effect on brain function. *Nat. Neurosci.* 3 91–96 10.1038/71163 10607401

[B71] Perry B. D. (2002). Childhood experience and the expression of genetic potential: what childhood neglect tells us about nature and nurture. *Brain Mind* 3 79–100 10.1023/A:1016557824657

[B72] Phan K. L. Fitzgerald D. A. Nathan P. J. Tancer M. E. (2006). Association between amygdala hyperactivity to harsh faces and severity of social anxiety in generalized social phobia. *Biol. Psychiatry* 59 424–429 10.1016/j.biopsych.2005.08.012 16256956

[B73] Pickering A. (2010). *The Cybernetic Brain: Sketches of Another Future*. Chicago, IL: University of Chicago Press.doi: 10.7208/chicago/9780226667928.001.0001

[B74] Ramsey J. D. Hanson S. J. Hanson C. Halchenko Y. O. Poldrack R. A. Glymour C. (2010). Six problems for causal inference from fMRI. *Neuroimage* 49 1545–1558 10.1016/j.neuroimage.2009.08.065 19747552

[B75] Rees C. (2008). The influence of emotional neglect on development. *Pediatr. Child Health*, 18 527–534 10.1016/j.paed.2008.09.003

[B76] Regier D. A. Kuhl E. A. Kupfer D. J. (2013). The DSM-5: classification and criteria changes. *World Psychiatry* 12 92–98 10.1002/wps.20050 23737408 PMC3683251

[B77] Robins E. Guze S. B. (1970). Establishment of diagnostic validity in psychiatric illness: its application to schizophrenia. *Am. J. Psychiatry* 126 107–111 10.1176/ajp.126.7.983 5409569

[B78] Rowlands M. (2010). *The New Science of the Mind: From Extended Mind to Embodied Phenomenology*. Cambridge, MA: MIT Press.doi: 10.7551/mitpress/9780262014557.001.0001

[B79] Seligman R. Choudhury S. Kirmayer L. J. (in press). “Locating culture in the brain and in the world: from social categories to the ecology of mind,” in *Handbook of Cultural Neuroscience* edsChiao J. Y. Turner R. Li S. Seligman R. (Oxford: Oxford University Press)

[B80] Shulman R. G. (2013). *Brain Imaging: What It Can (and Cannot) Tell Us about Consciousness*. Oxford: Oxford University Press 10.1093/acprof:oso/9780199838721.001.0001

[B81] Simpson H. B. (2012). The RDoC project: a new paradigm for investigating the pathophysiology of anxiety. *Depress. Anxiety* 29 251–252 10.1002/da.21935 22511360

[B82] Sporns O. (2013). The human connectome: origins and challenges. *Neuroimage* 80 53–61 10.1016/j.neuroimage.2013.03.023 23528922

[B83] Stanghellini G. Bolton D. Fulford W. K. (2013). Person-centered psychopathology of schizophrenia: building on Karl Jaspers’ understanding of patient’s attitude toward his illness. *Schizophr. Bull.* 39 287–294 10.1093/schbul/sbs154 23314193 PMC3576158

[B84] Strand M. (2011). Where do classifications come from? The DSM-III, the transformation of American psychiatry, and the problem of origins in the sociology of knowledge. *Theory Soc.* 40 273–313 10.1007/s11186-011-9142-8

[B85] Tiles M. (1993). The normal and pathological: the concept of a scientific medicine. *Br. J. Philos. Sci.* 44 729–742 10.1093/bjps/44.4.729

[B86] Todd M. Nystrom L. Cohen J. (2013). Confounds in multivariate pattern analysis: theory and rule representation case study. *Neuroimage* 77 157–165 10.1016/j.neuroimage.2013.03.039 23558095

[B87] Tretter F. Gebicke-Haerter P. J. (2009). Philosophy of neuroscience and options of systems science. *Pharmacopsychiatry* 42(Suppl.1) S2–S10 10.1055/s-0029-1215598 19434553

[B88] Turner R. (2002). Culture and the human brain. *Anthropol. Hum*. 26 167–172 10.1525/ahu.2001.26.2.167

[B89] Varela F. J. Thompson E. Rosch E. (1991). *The Embodied Mind: Cognitive Science and Human Experience*. Cambridge: MIT Press

[B90] Vul E. Harris C. Winkielman P. Pashler H. (2009). Puzzlingly high correlations in fMRI studies of emotion, personality, and social cognition. *Perspect. Psychol. Sci*. 4 274–290 10.1111/j.1745-6924.2009.01125.x 26158964

[B91] Wakefield J. C. (1992). Disorder as harmful dysfunction: a conceptual critique of DSM-III-R’s definition of mental disorder. *Psychol. Rev.* 99 232 10.1037/0033-295X.99.2.232 1594724

[B92] Wakefield J. C. (2007). The concept of mental disorder: diagnostic implications of the harmful dysfunction analysis. *World Psychiatry* 6 149 PMC2174594 18188432

[B93] Walter H. (2013). The third wave of biological psychiatry. *Front. Psychol.* 4:582 10.3389/fpsyg.2013.00582 PMC3763485 24046754

[B94] Wang C. Oyserman D. Liu Q. Li H. Han S. (2013). Accessible cultural mind-set modulates default mode activity: evidence for the culturally situated brain. *Soc. Neurosci.* 8 203–216 10.1080/17470919.2013.775966 23485156

[B95] Watts-English T. Fortson B. L. Gibler N. Hooper S. R De Bellis M. D. (2006). The psychobiology of maltreatment in childhood. *J. Soc. Issues* 62 717–736 10.1111/j.1540-4560.2006.00484.x

[B96] Weinberger D. R. Goldberg T. E. (2014). RDoCs redux. *World Psychiatry * 13: 36–38 10.1002/wps.20096 24497241 PMC3918012

[B97] Wiener N. (1948/1961). *Cybernetics; or, Control and Communication in the Animal and the Machine* 2nd Edn. Cambridge: MIT Press

[B98] Whooley O. (2014). Nosological reflections: the failure of DSM-5, the emergence of RDoC, and the decontextualization of mental distress. *Soc. Ment. Health*. 10.1177/2156869313519114

[B99] Wilson M. (1993). DSM-III and the transformation of American psychiatry: a history. *Am. J. Psychiatry* 150 399–410 8434655 10.1176/ajp.150.3.399

[B100] Zhang T. Y. Meaney M. J. (2010). Epigenetics and the environmental regulation of the genome and its function. *Annu. Rev. Psychol.* 61 439–466 C431–C433 10.1146/annurev.psych.60.110707.163625 19958180

